# Readmission after rectal resection in the ERAS-era: is a loop ileostomy the Achilles heel?

**DOI:** 10.1186/s12893-021-01242-y

**Published:** 2021-05-27

**Authors:** Johanna Van Butsele, Gabriele Bislenghi, André D’Hoore, Albert M. Wolthuis

**Affiliations:** Department of Abdominal Surgery, University Hospital Gasthuisberg, Leuven, Belgium

**Keywords:** Rectal resection, Readmission, Ileostomy, Risk factors, ERAS

## Abstract

**Background:**

Rectal resection surgery is often followed by a loop ileostomy creation. Despite improvements in surgical technique and development of enhanced recovery after surgery (ERAS) protocols, the readmission-rate after rectal resection is still estimated to be around 30%. The purpose of this study was to identify risk factors for readmission after rectal resection surgery. This study also investigated whether elderly patients (≥ 65 years old) dispose of a distinct patient profile and associated risk factors for readmission.

**Methods:**

This is a retrospective study of prospectively collected data from patients who consecutively underwent rectal resection for cancer within an ERAS protocol between 2011 and 2016. The primary study endpoint was 90-day readmission. Patients with and without readmission within 90 days were compared. Additional subgroup analysis was performed in patients ≥ 65 years old.

**Results:**

A total of 344 patients were included, and 25% (n = 85) were readmitted. Main reasons for readmission were acute renal insufficiency (24%), small bowel obstruction (20%), anastomotic leakage (15%) and high output stoma (11%). In multivariate logistic regression, elevated initial creatinine level (cut-off values: 0.67–1.17 mg/dl) (OR 1.95, p = 0.041) and neoadjuvant radiotherapy (OR 2.63, p = 0.031) were significantly associated with readmission. For ileostomy related problems, elevated initial creatinine level (OR 2.76, p = 0.021) was identified to be significant.

**Conclusion:**

Recovery after rectal resection within an ERAS protocol is hampered by the presence of a loop ileostomy. ERAS protocols should include stoma education and high output stoma prevention.

## Background

A defunctioning ileostomy is often created to optimize postoperative outcome after restorative rectal resection and to reduce the risk of anastomotic leakage [1, 2]. Enhanced recovery after surgery (ERAS) protocols were developed and implemented to improve postoperative recovery [3]. ERAS guidelines consist of pre-, peri- and postoperative evidence-based treatment measures aiming to reduce the number of complications and shorten the length of hospital stay [3–6]. Those measures consist among other things of early postoperative refeeding and mobilization, thromboembolic prophylaxis, oral carbohydrates preoperatively, opium-free anesthesia and avoidance of usage of nasogastric tubes. Despite all these efforts, 30- to 60-day readmission rates after restorative rectal resection are still estimated to be around 30% [7, 8]. Overall long-term morbidity rate after rectal resection has been reported to be 20–30% (mean follow-up time: 36–85 months) [9, 10]. Although a combination of efforts has led to improved recovery and shorter length of hospital stay, it is hypothesized that patients with a defunctioning ileostomy have a higher risk of acute renal insufficiency and of readmission. The aim of this study was to identify risk factors for readmission in patients after rectal resection and loop ileostomy creation.

## Methods

A retrospective database survey of prospectively collected data from patients who underwent rectal resection surgery within an ERAS-protocol over a 5-year period was conducted. In short, ERAS-protocol was implemented in 2009 and the following aspects were systematically used: preadmission counseling, no premedication, no nasogastric tube, multimodal perioperative analgesia, prevention of sodium and fluid overload, minimally-invasive approach with short incisions, prevention of hypothermia, thrombo-prophylaxis, routine postoperative mobilization, prevention of nausea and vomiting, early removal of catheters [11]. For rectal resections, all patients underwent mechanical bowel preparation as per hospital protocol. There was no systematic use of carbohydrate drinks (immune-nutritional therapy). Inclusion criteria were adult patients who underwent restorative proctectomy between 2011 and 2016. Exclusion criteria were patients who underwent rectal amputation with permanent colostomy, and urgent operations. Primary study endpoint was 90-day readmission. Two attending surgeons (ADH, AW) operated on these patients following the same principles. In general, ileostomies were performed in patients after neoadjuvant therapy, as per center protocol. Ileostomy-related problems were defined as all complications occurring because of the presence of an ileostomy. Complications such as parastomal skin problems, stoma necrosis (complete or partial), leakage caused by a low lying stoma, stenosis, soma bleeding, granuloma formation, prolapse, and parastomal hernia were recorded in the database. Loss of stoma output secondary to other causes was classified as ileostomy-related problem. High output stoma was defined as a stoma output exceeding 2000 ml/24 h. Acute renal insufficiency was defined as a decrease in renal function in the postoperative period, measured by an increase in serum creatinine or a decrease in urine output, or both. Anastomotic leakage was defined as a breach in a surgical join between two hollow viscera, with or without active leak of luminal contents. Readmission was defined as unanticipated need for hospitalization after rectal resection (index operation). Creatinine level was measured during hospital stay of the index operation. Initial creatinine level was the first value during hospital admission. Reference values were 0.51–0.95 mg/dl. Abnormal creatinine was defined as creatinine > 0.95 mg/dl. Additional subgroup analysis was performed in patients ≥ 65 years old. This study was ethically approved by The Research Ethics Committee UZ/KU Leuven (MP007786).

### Statistical analysis

Mann–Whitney U and Fishers exact tests were used to compare continuous/ordinal and categorical variables, respectively, between patients with and without readmission within 90 days. The discriminative ability (C-index) was reported for each of the considered predictors of readmission (0.5 = random prediction, 1 = perfect discrimination). A multivariable logistic regression model was obtained applying a backward selection strategy with p = 0.157 as critical p-value to stay in the model. The use of this critical value corresponds to using the Aikake Information Criterion for model selection. With this criterion we require that the increase in model χ^2^ has to be larger than two times the degrees of freedom. As an alternative, a stepwise selection procedure was used, yielding the same result. The prediction model obtained after applying a model building approach is overoptimistic, in the sense that it overestimates the future performance in new subjects. An optimism-corrected estimate of the performance was obtained using a bootstrap resampling procedure [12]. A similar approach was used to evaluate relations with the presence of an ileostomy problem within 90 days post discharge. Of note: time until readmission was not predicted, but readmission within 90 days. All analyses have been performed using SAS software, version 9.4 of the SAS System for Windows.

## Results

### Patient characteristics

A total of 344 patients who underwent rectal resection within an ERAS-protocol were included, 163 of which were older than 65 years old. Patient characteristics and operative details are shown in Tables 1 and 2. Mean age was 64 ± 11 years, whereas mean age in the elderly population was 73 ± 6 years. Older patients and the overall population showed a remarkably similar patient profile. Overall, only one third of the patients were female (32.9%). The majority of patients could be categorized in American Society of Anesthesiologists (ASA) category II (67.7%) and were treated with neoadjuvant therapy (68%). Sixty-seven percent (n = 231) of the patients received a loop ileostomy. Mean postoperative length of stay was 12 ± 9 days (median 9 (IQR 7–14) days). Overall readmission rate was 25% (85 out of 344 patients). Comparable rates of readmission were found in patients < 65 and ≥ 65 years old: 25% (45 out of 181) and 25% (40 out of 163), respectively. In univariate analysis, there was a significant difference in rate of treatment with neoadjuvant radiotherapy in the patient population older than 65 years old between the readmitted and non-readmitted group (30% vs. 9.8% respectively, p = 0.005). No difference was found in readmission rates between patients who did and did not receive a loop ileostomy. There were no patients lost to follow-up.

**Table 1:** Patient characteristics and operative details

Characteristic	Overall	No readmission	Readmission	p value
	n = 344	n = 259	n = 85	
Age (mean ± SD)	63.8 ± 11.4	63.9 ± 11	63.3 ± 12.7	0.876
Gender				
Male	231 (67.2%)	175 (67.6%)	56 (65.9%)	0.791
Female	113 (32.9%)	84 (32.4%)	29 (34.1%)	
Weight (mean ± SD)	77.8 ± 16.4	77.7 ± 16.3	78.1 ± 16.7	0.499
BMI (mean ± SD)	26.5 ± 4.9	26.4 ± 4.9	26.6 ± 4.6	0.517
ASA class				
I	31 (9%)	27 (10.4%)	4 (4.7%)	0.179
II	233 (67.7%)	168 (64.9%)	65 (76.4%)	
III	79 (23%)	63 (24.3%)	16 (18.8%)	
IV	1 (0.3%)	1 (0.4%)	0 (0%)	
Smoking behavior				
Never	186 (54.2%)	141 (54.7%)	45 (52.9%)	0.818
Stopped smoking	116 (33.8%)	85 (33%)	31 (36.5%)	
Actual smoker	41 (12%)	32 (12.4%)	9 (10.6%)	
Charlson comorbidity index (mean ± SD)	4.9 ± 2.1	4.9 ± 2.1	4.9 ± 2	0.785
Initial creatinine				
Abnormal	66 (19.2%)	45 (17.4%)	21 (24.7%)	0.154
Normal	278 (80.8%)	214 (82.6%)	64 (75.3%)	
Neoadjuvant therapy				
No	110 (32%)	87 (33.6%)	23 (27.1%)	0.061
Chemotherapy	10 (2.9%)	7 (2.7%)	3 (3.5%)	
Radiotherapy	32 (9.3%)	18 (7%)	14 (16.5%)	
Chemoradiotherapy	192 (55.8%)	147 (56.8%)	45 (52.9%)	
Mode of surgery				
Open	66 (19.2%)	50 (19.3%)	16 (18.8%)	0.615
Open converted	28 (8.1%)	19 (7.3%)	9 (10.6%)	
Laparoscopic	250 (72.7%)	190 (73.4%)	60 (70.6%)	
Additional surgery				
No	318 (92.4%)	241 (93.1%)	77 (90.6%)	0.480
Yes	26 (7.6%)	18 (7%)	8 (9.4%)	
Ileostoma				
No	113 (32.9%)	83 (32.1%)	30 (35.3%)	0.697
Already present	1 (0.3%)	1 (0.4%)	0 (0%)	
Newly placed	230 (66.9%)	175 (67.6%)	55 (64.7%)	
Duration surgery (h) (mean ± SD)	3.3 ± 0.9	3.3 ± 0.8	3.3 ± 1	0.844
Blood loss (dl) (mean ± SD)	2.7 ± 3.4	2.6 ± 3.4	2.9 ± 3.4	0.503
Length of stay (mean ± SD)	12.1 ± 9.3	12 ± 9.9	12.3 ± 7.1	0.104
Creatinine at discharge (mean ± SD)	0.9 ± 0.3	0.9 ± 0.3	0.9 ± 0.3	0.808

**Table 2:** Patient characteristics and operative details in patients > 65 years old

Characteristic	Age > 65 years	No readmission	Readmission	p value
	n = 163	n = 123	n = 40	
Age (mean ± SD)	73.1 ± 6.1	72.8 ± 6.2	74.1 ± 5.7	0.186
Gender				
Male	119 (73%)	90 (73.2%)	29 (72.5%)	1.000
Female	44 (27%)	33 (26.8%)	11 (27.5%)	
Weight (mean ± SD)	78.2 ± 15	78 ± 14.8	79 ± 15.8	0.399
BMI (mean ± SD)	27.2 ± 4.7	27.1 ± 4.7	27.8 ± 4.6	0.188
ASA class				
I	6 (3.7%)	6 (4.9%)	0 (0%)	0.497
II	103 (63.2%)	76 (61.8%)	27 (67.5%)	
III	54 (33.1%)	41 (33.3%)	13 (32.5%)	
IV				
Smoking behavior				
Never	83 (50.9%)	64 (52%)	19 (47.5%)	0.772
Stopped smoking	69 (42.3%)	50 (40.7%)	19 (47.5%)	
Actual smoker	11 (6.8%)	9 (7.3%)	2 (5%)	
Charlson comorbidity index (mean ± SD)	5.9 ± 1.8	5.8 ± 1.9	6 ± 1.5	0.173
Initial creatinine				
Abnormal	39 (23.9%)	26 (21.1%)	13 (32.5%)	0.199
Normal	124 (76.1%)	97 (78.9%)	27 (67.5%)	
Neoadjuvant therapy				
No	60 (36.8%)	49 (39.8%)	11 (27.5%)	0.005
Chemotherapy	3 (1.8%)	1 (0.8%)	2 (5%)	
Radiotherapy	24 (14.7%)	12 (9.8%)	12 (30%)	
Chemoradiotherapy	76 (46.6%)	61 (49.6%)	15 (37.5%)	
Mode of surgery				
Open	31 (19%)	25 (20.3%)	6 (15%)	0.676
Open converted	13 (8%)	9 (7.3%)	4 (10%)	
Laparoscopic	119 (73%)	89 (72.4%)	30 (75%)	
Additional surgery				
No	153 (93.9%)	115 (93.5%)	38 (95%)	1.000
Yes	10 (6.1%)	8 (6.5%)	2 (5%)	
Ileostoma				
No	39 (23.9%)	30 (24.4%)	9 (22.5%)	1.000
Already present	1 (0.6%)	1 (0.81%)	0 (0%)	
Newly placed	123 (75.5%)	92 (74.8%)	31 (77.5%)	
Duration surgery (h) (mean ± SD)	3.3 ± 0.9	3.3 ± 0.7	3.5 ± 1.1	0.820
Blood loss (dl) (mean ± SD)	2.6 ± 3	2.6 ± 3.2	2.5 ± 2.6	0.966
Length of stay (mean ± SD)	12.5 ± 8.3	12 ± 8.1	14.2 ± 8.9	0.142
Creatinine at discharge (mean ± SD)	1 ± 0.3	1 ± 0.3	1 ± 0.3	0.589

### Prediction of readmission

**Fig. 1:**
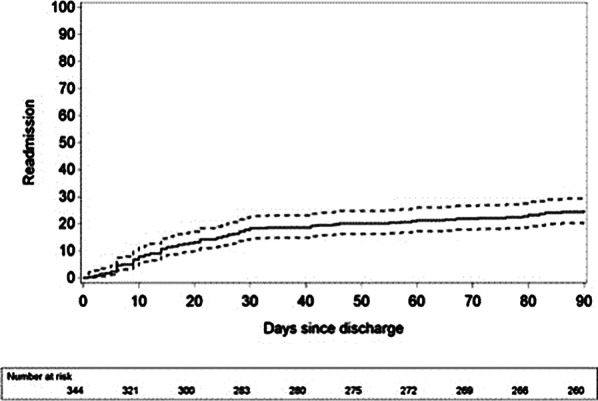
Readmission rate

Figure 1 shows that 18.3% (14.9–22.4%, 95% CI) of patients were readmitted within 30 days after discharge, 21.2% (17.7–25.4%, 95% CI) within 60 days after discharge and 24.7% (21.0–28.9%, 95% CI) within 90 days after discharge. Furthermore, mean duration of readmission was 9 ± 9 days.

Main reasons for readmission, together encompassing 70% of the cases were: acute renal insufficiency (24%), small bowel obstruction (20%), anastomotic leakage (15%) and high output stoma (11%) (Tables 3 and 4). Multivariate logistic regression analysis was used to determine which factors were associated with readmission. Abnormal initial creatinine and neoadjuvant radiotherapy were identified as significantly associated with readmission in the overall population (resp. OR = 1.95, p = 0.041 and OR = 2.63, p = 0.031) (Table 5).

**Table 3:** Reasons for readmission

Variable	Overall	No readmission	Readmission	p value
	n = 344	n = 259	n = 85	
Any complication				
No	225 (65.4%)	173 (66.8%)	52 (61.2%)	0.360
Yes	119 (34.6%)	86 (33.2%)	33 (38.8%)	
Number of complications				
0	225 (65.4%)	173 (66.8%)	52 (61.2%)	0.221
1	81 (23.6%)	54 (20.9%)	27 (31.8%)	
2	26 (7.6%)	22 (8.5%)	4 (4.7%)	
3	5 (1.5%)	5 (1.9%)	0 (0%)	
4	6 (1.7%)	4 (1.5%)	2 (2.4%)	
5	1 (0.3%)	1 (0.4%)	0 (0%)	
Anastomotic leakage				
No	320 (93%)	241 (93%)	79 (92.9%)	1.000
Yes	24 (7%)	18 (7%)	6 (7.1%)	
Postoperative bleeding				
No	340 (98.8%)	255 (98.5%)	85 (100%)	0.576
Yes	4 (1.2%)	4 (1.5%)	0 (0%)	
Postoperative ileus				
No	302 (87.8%)	226 (87.3%)	76 (89.4%)	0.704
Yes	42 (12.2%)	33 (12.7%)	9 (10.6%)	
SSI type 1 wound infection				
No	338 (98.3%)	256 (98.8%)	82 (96.5%)	0.163
Yes	6 (1.7%)	3 (1.2%)	3 (3.5%)	
Urinary retention				
No	321 (93.3%)	240 (92.7%)	81 (95.3%)	0.466
Yes	23 (6.7%)	19 (7.3%)	4 (4.7%)	
UTI, urological infection				
No	330 (95.9%)	249 (96.1%)	81 (95.3%)	0.754
Yes	14 (4.1%)	10 (3.9%)	4 (4.7%)	
Cardiac complication				
No	338 (98.3%)	256 (98.8%)	82 (96.5%)	0.163
Yes	6 (1.7%)	3 (1.2%)	3 (3.5%)	
Lung complication				
No	334 (97.1%)	251 (96.9%)	83 (97.7%)	1.000
Yes	10 (2.9%)	8 (3.1%)	2 (2.4%)	
Renal complication				
No	333 (96.8%)	250 (96.5%)	83 (97.7%)	1.000
Yes	11 (3.2%)	9 (3.5%)	2 (2.4%)	
Catheter acquired infection				
No	328 (95.4%)	246 (95%)	82 (96.5%)	0.769
Yes	16 (4.7%)	13 (5%)	3 (3.5%)	
High output stoma				
No	325 (94.5%)	247 (95.4%)	78 (91.8%)	0.271
Yes	19 (5.5%)	12 (4.6%)	7 (8.2%)	
Small bowel obstruction				
No	341 (99.1%)	256 (98.8%)	85 (100%)	1.000
Yes	3 (0.9%)	3 (1.2%)	0 (0%)	
Ileostomy problem				
No	314 (91.3%)	238 (91.9%)	76 (89.4%)	0.508
Yes	30 (8.7%)	21 (8.1%)	9 (10.6%)

**Table 4:** Reasons for readmission in patients > 65 years old

Variable	Age > 65 years	No readmission	Readmission	p value
	n = 163	n = 123	n = 40	
Any complication				
No	101 (62.0%)	79 (64.2%)	22 (55%)	0.350
Yes	62 (38%)	44 (35.8%)	18 (45%)	
Number of complications				
0	101 (62%)	79 (64.2%)	22 (55%)	0.329
1	41 (25.2%)	27 (22%)	14 (35%)	
2	14 (8.6%)	12 (9.8%)	2 (5%)	
3	2 (1.2%)	2 (1.6%)	0 (0%)	
4	5 (3.1%)	3 (2.4%)	2 (5%)	
5				
Anastomotic leakage				
No	157 (96.3%)	118 (95.9%)	39 (97.5%)	1.000
Yes	6 (3.7%)	5 (4.1%)	1 (2.5%)	
Postoperative bleeding				
No	161 (98.8%)	121 (98.4%)	40 (100%)	1.000
Yes	2 (1.2%)	2 (1.6%)	0 (0%)	
Postoperative ileus				
No	141 (86.5%)	105 (85.4%)	36 (90%)	0.598
Yes	22 (13.5%)	18 (14.6%)	4 (10%)	
SSI type 1 wound infection				
No	159 (97.6%)	121 (98.4%)	38 (95%)	0.253
Yes	4 (2.5%)	2 (1.6%)	2 (5%)	
Urinary retention				
No	147 (90.2%)	110 (89.4%)	37 (92.5%)	0.763
Yes	16 (9.8%)	13 (10.6%)	3 (7.5%)	
UTI, urological infection				
No	157 (96.3%)	119 (96.8%)	38 (95%)	0.636
Yes	6 (3.7%)	4 (3.3%)	2 (5%)	
Cardiac complication				
No	158 (96.9%)	121 (98.4%)	37 (92.5%)	0.095
Yes	5 (3.1%)	2 (1.6%)	3 (7.5%)	
Lung complication				
No	156 (95.7%)	118 (95.9%)	38 (95%)	0.681
Yes	7 (4.3%)	5 (4.1%)	2 (5%)	
Renal complication				
No	155 (95.1%)	117 (95.1%)	38 (95%)	1.000
Yes	8 (4.9%)	6 (4.9%)	2 (5%)	
Catheter acquired infection				
No	155 (95.1%)	117 (95.1%)	38 (95%)	1.000
Yes	8 (4.9%)	6 (4.9%)	2 (5%)	
High output stoma				
No	152 (93.3%)	117 (95.1%)	35 (87.5%)	0.140
Yes	11 (6.8%)	6 (4.9%)	5 (12.5%)	
Small bowel obstruction				
No	163 (100%)	123 (100%)	40 (100%)	
Yes				
Ileostomy problem				
No	144 (88.3%)	111 (90.2%)	33 (82.5%)	0.254
Yes	19 (11.7%)	12 (9.8%)	7 (17.5%)

**Table 5:** Multivariate prediction of 90-day readmission: stepwise multivariate logistic regression model

	Odds ratio (95% CI)	p value
ASA		
ASA 2		0.049
ASA 3–4	3.8 (1.1–13.1)	0.036
	2.3 (0.6–8.6)	0.228
Initial creatinine		
Abnormal	2 (1.0–3.7)	0.041
Neoadjuvant therapy		0.134
Chemotherapy	1.8 (0.4–7.5)	0.443
Chemoradiotherapy	1.1 (0.6–1.9)	0.831
Radiotherapy	2.6 (1.1–6.3)	0.031

### Prediction of ileostomy problems

Patients who suffered from an ileostomy-related problem were older than patients who did not: mean age 68 ± 11 years versus 63 ± 11 years, respectively (p = 0.025). Abnormal initial creatinine value (OR = 2.76, p = 0.021) was determined as risk factor for development of ileostomy problems (Table 6).

**Table 6:** Multivariate prediction of 90-day ileostomy problem: stepwise multivariate logistic regression model

	Odds ratio (95% CI)	p value
Initial creatinine		
Abnormal	2.8 (1.7–6.5)	0.021
Mode of surgery		0.1475
Laparoscopic	0.4 (0.2–1.0)	0.052
Open converted	0.6 (0.1–2.5)	0.454
Ileostomy problem		
Yes	2.6 (0.9–7.6)	0.088

## Discussion

This study shows that the readmission rate after rectal resection was 25%, and most readmissions occurred within 30 days after discharge. These findings are in line with the literature (Table 7) [6–8, 13–22]. Abnormal initial creatinine and neoadjuvant therapy were identified as significantly associated with readmission. Moreover, most patients were readmitted because of acute renal insufficiency secondary to ileostomy-related problems. In a similar study, unplanned hospital readmission following ileostomy was 29%. Also, renal impairment at discharge was the most important risk factor to predict readmission [23]. In another recent study, Fielding et al. found that postoperative renal impairment more frequently occurred in patients with a diverting ileostomy. Moreover, ileostomy formation was independently associated with kidney injury, and continued to have an impact, even after stoma closure [24]. Another study from the NSQIP dataset by Kim et al. showed that patients with postoperative renal impairment were much more likely to be readmitted after ileostomy creation [25]. O’Connell et al. identified surgical site infection (SSI) and stoma formation as significant risk factors for readmission in a study with a comparative sample size [26]. This can be attributed to the fact that firstly, SSI rate was much lower in our population (1.7% versus almost 10%) and secondly, the conclusion concerning stoma formation in the study by O’Connell et al. was based on seven cases (4/31 in the readmission group, 3/215 in the no-readmission group) [26]. We also observed an increased readmission risk after stoma formation (7/85 in the readmission group, 12/259 in the no-readmission group), although this was not statistically significant. It has already been shown that patients who received a stoma after colorectal resection are more likely to be readmitted to the hospital [7, 27, 28]. Many factors associated with readmission like age and past medical history are not prone to modification. In those high-risk cases, reduction of readmission should be attempted through adequate patient selection and preoperative optimization. The implementation of ERAS guidelines may play a major role in that matter. However, our study shows that despite the implementation of ERAS measures, the risk of readmission remains high in the patient population treated with a loop ileostomy. Therefore, efforts should be made to further reduce this risk. Shaffer et al. observed a 58% reduction of readmission rates and a more than 80% reduction in readmission-related costs after implementation of a specific patient follow-up program [29]. A similar program set up by Nagle et al. also resulted in a significant decrease of readmissions (15.5% to 0%) [30]. Shah et al. and Hardiman et al. obtained similar results using an enhanced recovery protocol and a patient self-care checklist, respectively [14, 17]. Iqbal et al. even found that a lack of a social worker involvement in planning for discharge is associated with the highest risk of readmission of all factors analyzed in their series (OR 5.15) [20]. These data suggest that patient guidance and monitoring could be of utmost importance in the attempt to reduce readmission rates and associated costs in ileostomy patients. The fact that in the present study, readmission rate was equal in both age categories is in line with what was reported by Kandagatla et al. [31]. It could be explained that nowadays overall health status, rather than age, influences the postoperative course the most. We also observed that readmission rate did not depend on surgical approach, meaning that presence of an ileostomy was a more important factor. The strengths of our study include a homogenous patient population, consisting of all rectal resection patients and our strict inclusion and exclusion criteria. Our study is unique as it only involves patients who underwent rectal resection and follow-up time is much longer than usual (90-day readmission).

**Table 7:** Overview of the literature

	Sample size	Readmission rate (%)	Reason readmission	Risk factors	Protective factors
Li et al. 2017 [13]	1267	12.9	Infections (3.4%) Small bowel obstruction/ileus (3.3%) Dehydration (38.3%)	Cardiovascular factors (OR 2.0) Renal comorbidity (OR 2.9) Preoperative chemo/radiotherapy (OR 4.0) Laparoscopic approach (OR 1.7) Longer operative time (OR 1.2) Due to dehydration: Chemo/radiotherapy (OR 4.7) Laparoscopic approach (OR 2.6)	Cancer diagnosis (OR 0.2)
Fish et al. 2017 [7]	407	28	Dehydration (42%) Intraperitoneal infections (33%) Extraperitoneal infections (29%)	Clavien-Dindo complication grade 3 to 4 (OR 6.7) Charlson comorbidity index (OR 1.4 per point) Loop stoma (OR 2.2)	Longer length of stay (OR 0.5) Age 65 years or older (OR 0.4)
Shah et al. 2017 [14]	707	12		Ileostomy	Enhanced recovery protocol
Wood et al. 2017	2876	8.2	Ileus and nausea/vomiting (26.1%) Intra-abdominal ascess (23.9%) SSI (11.5%)	Rectal surgery (OR 1.89) Stoma formation (OR 1.34) Reoperation during first admission (OR 4.60)	
Justiniano et al. 2018 [8]	262	30	Dehydration (37%)		
Hayden et al. 2012	154	20.1		Use of anti-diarrheals Neoadjuvant therapy	
Messaris et al. 2012 [16]	603	16.9	Dehydration (43.1%)	Laparoscopic approach Lack of epidural aneshtesia Preoperative use of sterois Postoperative use of diuretics	
Hardiman et al. 2016 [17]	430	26			
Charak et al. 2018 [18]	99	36	Dehydration (39%) Infection (33%) Obstruction (3%)		
Grahn et al. 2018	100	19.6–20.4	Dehydration (5.9–8.2%) Acute renal failure events (3.9–10.2%)	Weekend discharges to home (OR 4.5)	
Iqbal et al. 2018 [20]	86	26		Preoperative steroid use History of diabetes History of depression Lack of hospital social worker or postoperative ostomy education Presence of complications after the index procedure	
Paquette et al. 2013 [21]	201	17		Age greater than 50 IPAA	
Chen et al. 2018 [22]	8064	20.1		ASA class III Female sex IPAA Age > 65 Shortened length of stay ASA class I to II with IBD Hypertension	

The retrospective nature of our study is a potential limitation, as well as the fact that it is a single center study which yielded a limited number of patients. For patients treated within an ERAS protocol, length of hospital stay was rather long. This might be due to the fact that patient’s preference regarding discharge plays a role. Unfortunately, data regarding fit for discharge and actual discharge were not available, and could be considered a drawback. Furthermore, patients who were readmitted in outside hospitals were not taken into account and manual analysis of patient files and the use of a coding system was subject to human error. Another limitation of the present study was the lack of information on frailty in older patients and the fact that perioperative fluid balance was not exactly known. Prevention and patient education are key features to avoid readmission secondary to dehydration and ileostomy-related problems. Currently, a patient-centered protocol and follow-up to detect complications at an early stage via teleconsulting by a specialist nurse are under investigation at our department [32].

## Conclusion

Readmission after rectal resection in the ERAS-era occurs in 25% of the cases. Most readmissions occur within 30 days after index hospitalization and acute renal insufficiency is frequently associated with readmission. Future patient-education initiatives should be used in conjunction with ERAS guidelines to reduce postoperative readmission.

## Data Availability

The dataset analysed during the current study is available from the corresponding author on reasonable request.

## Abbreviations

ASA: American Society of Anesthesiologists
ERAS: Enhanced recovery after surgery
SSI: Surgical site infection
OR: Odds ratio
